# Investigating the Probiotic Properties and Antimicrobial Activity of Lactic Acid Bacteria Isolated from an Iranian Fermented Dairy Product, Kashk

**DOI:** 10.3390/foods11233904

**Published:** 2022-12-03

**Authors:** Bahareh Saboori, Fakhri Shahidi, Sara Hedayati, Ali Javadmanesh

**Affiliations:** 1 Department of Food Science and Technology, Faculty of Agriculture, Ferdowsi University of Mashhad (FUM), Mashhad 9177948978, Iran; 2 Nutrition Research Center, School of Nutrition and Food Sciences, Shiraz University of Medical Sciences, Shiraz 7193635899, Iran; 3 Department of Animal Science, Faculty of Agriculture, Ferdowsi University of Mashhad (FUM), Mashhad 9177948978, Iran

**Keywords:** kashk, functional foods, lactic acid bacteria, molecular identification, probiotic properties

## Abstract

In the present study, kashk samples were collected from two regions of Iran, the Fars (Abadeh) and Razavi Khorasan (Kalat) provinces. Fifteen bacteria were isolated and physiological and biochemical assays were performed. After identification to the genus level, eight isolates were identified as lactic acid bacteria (LAB) and subjected to molecular identification and probiotic properties assays. The results revealed that the isolates were *Enterococcus faecium* KKP 3772 (KF1), *Enterococcus faecium* C1 (KF2), *Pediococcus pentosaceus* H11 (KF3), *Pediococcus pentosaceus* VNK-1 (KK4), *Lactococcus lactis* RSg (KK1), *Enterococcus faecalis* P190052 (KK2), *Enterococcus mundtii* CECT972T (KK3), and *Lactiplantibacillus plantarum* PM411 (KK5). Only the numbers of *L. lactis* RSg (KK1) and *Lpb. Plantarum* PM411 (KK5) decreased to below 9 Log CFU/mL after acidic conditions (pH = 3) and showed weak antibacterial activity. *Enterococcus mundtii* CECT972T (KK3) and *E. faecium* C1(KF2) were highly susceptible to bile salts, while *P. pentosaceus* VNK-1 (KK4) and *P. pentosaceus* H11 (KF3) showed the highest resistance. All of the isolates were resistant to tetracycline and sensitive to chloramphenicol and gentamicin. The antimicrobial activity of *P. pentosaceus* VNK-1 (KK4) and *P. pentosaceus* H11 (KF3) was higher than other isolates and consequently, their inhibition zones were larger. The adhesion capabilities of LAB isolates to intestinal epithelial cells were evaluated by examining the auto-aggregation factor and cell surface hydrophobicity. The highest and lowest cell surface hydrophobicity and auto-aggregation were obtained from *P. pentosaceus* VNK-1 (KK4) and *E. mundtii* CECT972T (KK3), respectively. In general, *P. pentosaceus* VNK-1 (KK4) and *P. pentosaceus* H11 (KF3) have shown better probiotic properties as compared to other isolates.

## 1. Introduction

Kashk is a fermented dairy product that originated from the Middle East and is available in circular, oval, or conical shapes. The traditional product is primarily produced using raw sheep milk. Furthermore, it can be produced using by-products from cheese making, such as buttermilk, which are fermented, salted, and dried. Kashk is supplied in both liquid (industrial) and dried (traditional) forms. Industrial liquid kashk is manufactured from concentrated yogurt or dry kashk and should be stored in a refrigerator while the dried kashk can be kept at room temperature for a long time without spoilage or reduction in nutrients. Generally, kashk contains large amounts of proteins, B complex vitamins, minerals such as calcium, potassium, and organic acids (such as lactic acid) [1]. The properties of kashk, such as taste, aroma, and texture, depend on several factors, predominantly the enzymatic activities of lactic acid bacteria (LAB) as the main microbiota in dairy products [2].

Kashk is a rich source of beneficial LAB, particularly probiotic bacteria. LAB are a heterogeneous group of Gram-positive, catalase-negative, non-mobile, and non-spore-forming bacteria that are able to produce lactic acid. LAB are categorized in the phylum Firmicutes, class Bacilli or Lactobacillus [3,4]. These microorganisms have complex nutritional requirements for amino acids and vitamins (especially B-group vitamins) because they have lost much of their biosynthetic potential in the process of evolution. Consequently, they only grow in nutrient-rich environments such as plants, fermented foods (dairy products, fermented vegetables, etc.), soil, lakes, etc. Furthermore, LAB are frequently observed in the resident microbiota of the genitourinary tract and the gastrointestinal tract (GIT) of the vertebrates [5].

LAB play a significant role in preservation and fermentation processes. Therefore, they are the predominant microbiota of fermented products [6]. These beneficial bacteria are among the most important groups of industrial microorganisms and have wide applications in the food, chemical, cosmetic, and medicine industries [7,8]. LAB show a large set of technological and functional properties, which could enhance microbial safety and improve the sensory attributes of various foods. LAB also produce various antimicrobial substances, including organic acids, bacteriocins, hydrogen peroxide (H_2_O_2_), and diacetyl, which prevent the growth of many foodborne pathogens and spoilage microorganisms [9].

Probiotic is a Greek word where pro means “for” and bios means “life”, and it contrasts with the word antibiotic which is derived from anti (against) and bios (life). The most accepted definition of probiotics is live microorganisms, which confer health effects on the host when consumed in adequate amounts [10,11]. Acid and bile salts tolerance are two essential properties of probiotics, showing the ability of these microorganisms to survive during passing through the digestive tract, with resistance to the acidic environment of the stomach and bile salts of the small intestine [12]. 

In recent decades, probiotics have attracted growing interest because of their numerous health benefits. Recent studies have shown the beneficial therapeutic effects of probiotics in the prevention and treatment of diseases, such as infant diarrhea, antibiotic-related diarrhea, *Helicobacter pylori* infection, inflammatory bowel diseases (IBD), cancers, urinary tract infections (UTIs), autism, Parkinson disease, and reproductive tract infections (RTIs) [10,13].

Other benefits of probiotics include improvement in lactose intolerance, lowering cholesterol, improving nutritional intake, reducing side effects of antibiotics, improving digestion, and increasing resistance to infections [14]. Although, there are several ways to transfer probiotics to the human digestive system, one of the best ways is to consume fermented dairy foods and drinks such as kashk, kefir, yogurt, and cheese which contain these bacteria. Considering the important role of dairy products and probiotics in human health, this research was conducted with the aim of LAB isolation from two types of local Iranian kashk and investigation of their probiotic properties.

## 2. Materials and Methods

### 2.1. Screening of LAB

Nine kashk samples were collected from each region (Abadeh in Fars province and Kalat in Razavi Khorasan province) and eight LAB strains were isolated from kashk samples and were analyzed to determine their probiotic potential. 

In order to isolate lactobacilli, the samples were cultured on MRS agar medium and plates were incubated at 37 °C for 24–72 h in the presence of 0.03% carbon dioxide. In addition, to isolate streptococci, lactococci, and enterococci, the samples were grown on M17 agar medium and enumerated after 37 °C for 36–48 h anaerobically [15].

Bacterial colonies were identified based on the morphological (shape, color, and size), physiological (growth at 10, 40, and 45 °C, and growth at 2, 4, and 6.5 sodium chloride concentrations) and biochemical (catalase production, oxidase activity, and arginine hydrolysis) characteristics [2].

After the culturing of pure colonies, the genomic DNA of the bacteria was extracted. Identification of the microorganisms was performed by 16S rRNA gene sequencing as an accurate and reliable procedure. PCR amplification of approximately 1400 bp from 16S rRNA gene from the bacterial isolates was performed using the forward primer 27FYM (5′-AGAGTTTGATYMTGGCTCAG-3′) and the reverse primer 1492R (5′-GGTTACCTTGTTACGACTT-3′) [15]. PCR reactions were completed in a total volume of 25 µL and the reaction mixture contained 16.5 µL PCR-grade water, 2.5 µL 10× PCR buffer, 2 µL dNTPs (200 nM), 1.5 µL MgCl_2_ (25 mM), 0.2 µL Taq polymerase (1 unit), 2 µL primer (5 pM), and 1.5 µL DNA (50 ng). The PCR temperature cycling conditions were as follows: first step: 95 °C for 5 min, one cycle; followed by 35 cycles of the second step: 94 °C for 30 s, 50 °C for 45 s, 72 °C for 2 min; followed by the third step: 72 °C for 10 min, one cycle [16].

First, 1% agarose gel was prepared in a 1X TBE buffer (Tris, boric acid, EDTA). After the agarose has cooled, DNA green viewer was used to observe the bands under UV light. Electrophoresis was performed at a voltage of 95 v and a time of 45 min. Then, the gel was visualized in the GelDoc (gel documentation) system [17].

The PCR products from the 16S rRNA gene amplification of isolates were purified and sequenced in Macrogen (Seoul, South Korea). DNA sequencing was performed by using cycle extension in an ABI 373 DNA sequencer (Applied Biosystems, Foster City, CA, USA) using primer 27FYM. On average, 1100 bp were taken per sequence, which were then compared with sequences in the GenBank database by the BLAST program (http://www.ncbi.nlm.nih.gov, accessed on 20 October 2022) [17].

### 2.2. Assessment of Probiotic Properties

#### 2.2.1. Cell Survival in Simulated Digestive System

First, the LAB isolates were cultured in MRS broth and then incubated in 37 °C for 18–24 h anaerobically. Subsequently, the suspension was centrifuged (6000× *g*, 10 min, 4 °C) and the supernatant was removed. The collected cells were washed twice using sterile Phosphate-buffered saline (PBS) buffer (adjusted pH 2 and 3). Aliquots of microbial cells were cultured on MRS agar and then incubated at 37 °C for 0, 1, 2, and 3 h under anaerobic conditions. Finally, the quantity of viable bacteria was stated as colony formation unit per milliliter (CFU/mL).

To assess the survival of LAB isolates in the simulated conditions of the digestive system, overnight cultures were prepared from the desired isolates and then centrifuged at 8000× *g* for 5 min at 4 ℃. The pellet was washed with 50 mM PBS (pH 6.5) and then dissolved in 3 mL of PBS buffer. A 1 mL aliquot of the isolate comprising 9 log CFU/mL of bacteria was mixed with 9 mL of simulated gastric fluid which contained NaCl 125 mM, NaHCO_3_ 45 mM, KCl 7 mM, pepsin (3 gr/L) at pH = 2.5. The mixture was incubated at 37 °C for 3 h. Then the suspension was centrifuged at 3000× *g* for 10 min, and the supernatant was removed. The pellet was washed with PBS two times and resuspended in simulated intestinal fluid at pH = 8.0 which contained bile salt 0.15% *w*/*v*, pancreatin 0.1% *w*/*v*, and incubated at 37 ℃ for 3 h. After that, the number of viable bacteria was counted and reported as Log CFU/mL [18].

#### 2.2.2. Bile Salts Tolerance

To determine the bile salts tolerance of LAB, each bacterial strain (2% *v*/*v*) was cultured in MRS broth medium with different levels (0.3, 0.5, and 1% *w*/*v*) of bile salts. The culture without bile salt was considered the control culture. The cultures were incubated at 37 °C for 24 h, and then the absorbance was measured with a spectrophotometer at 560 nm (A 560) and compared with the control culture [19] 

#### 2.2.3. Antibiotic Susceptibility

Antibiotic susceptibility to 16 antibiotic agents was investigated: streptomycin (10 µg), colistin (10 µg), cefepime (30 µg), vancomycin (30 µg), sulfamethoxazole (2 µg), tetracycline (30 µg), chloramphenicol (30 µg), fosfomycin (200 µg), kanamycin (30 µg), gentamycin (10 µg), neomycin (30 µg), ceftriaxone (15 µg), cefixime (15 µg), erythromycin (15 µg), ciprofloxacin (5 µg), ampicillin (10 µg), and cephalexin (30 µg). The strains were cultured with a concentration of 0.5 McFarland in MRS agar. Afterward, antibiotic discs with a certain concentration were placed on the surface of the plate at a distance of 2–4 cm and kept at ambient temperature for 15 min. The Petri dishes were incubated for 24 h at 37 ℃. According to the recommendations of the Clinical and Laboratory Standards Institute (CLSI), the inhibition zones were measured and categorized as sensitive or resistant [20].

#### 2.2.4. Antibacterial Activity Assay

The LAB were cultured in MRS broth and then the overnight cultures were centrifuged at 6000× *g* for 10 min. at 4 ℃. The supernatant was separated and divided into 4 groups: (1) heat treatment (boiling for 10 min), (2) neutralization to pH 7 with NaOH, (3) treatment with catalase (0.5 mg/mL), (4) control (no treatment). Afterward, the supernatants were filter sterilized (0.22 µm), and 100 µL of each supernatant were transferred to the wells bored in agar plates and inoculated with 1% (*v*/*v*) overnight cultures of indicator pathogens, *Escherichia coli* ATCC 25922, *Staphylococcus aureus* ATCC 25923, *Pseudomonas aeruginosa* ATCC 27853, and *Salmonella typhimurium* PTCC 1609. The plates were incubated at 37 °C for 24 h, and the diameters (mm) of inhibition zones were measured [21]. 

#### 2.2.5. Cell Surface Hydrophobicity

Cell surface hydrophobicity assay was carried out following the method developed by Hashemi et al. (2014) [22]. The LAB were grown in MRS broth at 37 ℃ for 18–24 h anaerobically and then centrifuged (5000× *g*, 15 min). The pellets were washed with phosphate buffer and suspended in 0.1 M KNO_3_ at pH 6.2 to an approximate concentration of 10^8^ CFU/mL. Afterward, the absorbance of the bacterial suspension was measured at 600 nm (A_0_). The cell suspension (3 mL) was then mixed with xylene (1 mL), kept at ambient temperature for 10 min., and vortexed for 2 min. The aqueous phase was collected after 20 min of incubation at ambient temperature, and the absorbance was detected at 600 nm (A_1_). The cell surface hydrophobicity (%) was calculated by the following equation [23]:

(1 − A_1_/A_0_) × 100

#### 2.2.6. Auto-Aggregation Assay

The overnight cultures were centrifuged at 5000× *g* for 10 min at 4 °C. The pellets were washed with PBS twice followed by resuspension in the same buffer and the OD_600_ was measured immediately (A_0_). The mixture was incubated at 37 °C for 2 h and the OD_600_ was measured again (A_t_). Auto-aggregation (%) was calculated by the following equation [24]:

$$Auto-aggregation\;\%=\left[\frac{Abs_{initial}-Abs_{final}}{Abs_{initial}}\right]\times 100$$

### 2.3. Statistical Analysis

All measurements were performed in triplicate and the analysis was carried out with SPSS-20 statistical software. The differences within mean values were determined by the Duncan test at a 95% confidence level (*p* < 0.05).

## 3. Results and Discussion

### 3.1. Identification of Isolates

After purifying, 15 isolates were obtained and examined by morphological and biochemical identification. The preliminary assays were: Gram staining and catalase activity. Afterward, 8 g positive and catalase-negative strains were selected as potential LAB. The morphology of the isolates was examined by using a light microscope, and the cocci and bacillus-shaped isolates were selected for further assays. According to the results obtained from physiological, morphological, and biochemical assays, bacteria were classified based on their similarities and differences. Eight selected isolates were identified to genus level, based on biochemical assays: Group 1: Homofermentative bacilli that grew at 10 °C and pH = 4.4, but were unable to hydrolyze arginine, this group was identified as homofermentative lactobacilli; Group 2: Gram-positive, catalase-negative, homofermentative cocci with tetrad arrangement and unable to hydrolyze arginine. They grew at 10 °C, but not at 45 °C and pH = 9.6, so were considered pediococcus species; Group 3: The isolates of *Lactococcus* that could grow at 10 °C and hydrolyze arginine; Group 4: Gram-positive, catalase-negative and homofermentative cocci which grew at both 10 and 45 °C, in the presence of 6.5% of NaCl at pH = 9.6 were identified as Enterococcus spp. (Table 1) [17]. Therefore, the identified isolates included Lactobacillus, Pediococcus, Lactococcus, and *Enterococcus* species.

The results of 16S rRNA gene analysis showed that the bacterial isolates were *Enterococcus faecium* KKP 3772 (KF1), *Enterococcus faecium* C1 (KF2), *Pediococcus pentosaceus* H11(KF3), *Lactococcus lactis* Rsg (KK1), *Enterococcus faecalis* P190052 (KK2), *Enterococcus mundtii* CECT972T( KK3), *Pediococcus pentosaceus* VNK-1 (KK4), and *Lpb. plantarum* PM411 (KK5) (Table 2). Among these bacteria, the first three isolates were obtained from Abadeh (Fars) kashk samples and the others were isolated from Kalat kashk samples (Razavi Khorasan). *Lactococcus lactis* is an anaerobic homofermentative bacterium that produces large amounts of lactic acid (L^+^) during growth and metabolic activity. This organism is also able to produce lactic acid (D^-^) at a lower pH, and this ability is one of the reasons for the wide application of *Lactococcus lactis* in dairy industries [25]. Edalatian et al. (2012), stated that the Lighvan cheese microbiota comprises a consortium of Enterococci, including *Enterococcus faecalis* and *Enterococcus faecium* species as the dominant bacteria. The cause of the large population of Enterococcus bacteria in ripened Lighvan cheese is their tolerance to high salt concentrations and low pH. In addition to Enterococci, *Lpb. plantarum* was found in high concentrations in Lighvan cheese [17].

*Enterococcus* species are present in many fermented milk products. These bacteria have positive effects on the quality of Roquefort cheeses and increase the growth in other LAB. Specific strains of *Enterococcus* have shown probiotic potential such as resistance to a wide range of pH and temperature, bacteriocin production, etc. [26].

### 3.2. Cell Survival in Low pH Conditions, Simulated Digestive System, and Bile Salts

According to the definition by the World Health Organization (WHO), probiotics must be consumed in sufficient amounts (at least 10^7^ CFU/mL) to show their beneficial effects. Conditions such as the acidic pH of the environment can hinder metabolism and decrease the growth and survival of LAB [27]. Acids in the human stomach destroy biomolecules such as fatty acids, proteins, vitamins, and nucleic acids. Stomach cells secrete about 2 L of acidic gastric juices per day, which create strict conditions for the survival of microorganisms that pass through the stomach. The results of evaluating the resistance of lactic acid isolates to acidic conditions (pH 2 and 3) and gastric juices are presented in Table 3.

Different trends were observed in the survival of the isolates in acidic pH. As shown in Table 3, incubation at pH 2 for 3 h, reduced the bacterial growth by 3 to 6 logarithmic cycles. While the isolates remained unchanged at pH 3, except *Lactococcus lactis*, which showed about 1.5 logarithmic cycle loss in the cell viability after 3 h of incubation. The survival of LAB strains in the range of pH 2 to 4 is an essential factor to perform as potential probiotics [28,29].

In agreement with our results, Ding and Shah (2007) investigated the effect of pH ≥ 2.5 on the reduction of 8 different species of Lactobacillus. The findings indicated the high sensitivity of bacterial cells to low pH [30]. In another study, Parente et al. (2010), examined the activity of two enzymes, arginine deaminase, and glutamate decarboxylase, in *Lpb. plantarum*, when exposed to different concentrations of acid. They found that the activity level of two enzymes increased with the increase in acid concentration; however, this increase in activity level had a downward trend at pH ≤ 3 [31].

F_1_F_0_-ATPase is one of the main mechanisms to protect Gram-positive microorganisms against acidic conditions. Other factors, such as growth medium composition, cell membrane composition, nutritional compounds, and type of microorganism can affect pH resistance [32]. Azat et al. investigated the pH resistance of LAB isolated from traditionally fermented Xinjiang cheese and stated that all tested isolates were tolerant to acidic and bile salt conditions, as the viable population of all strains was found to be more than 10^6^ CFU/mL after incubation at pH 3 for 3 h [33]. 

To investigate the probiotic potential of an isolate, it is essential to assess its tolerance to bile salts. Bile is a digestive secretion that breaks down large lipid droplets into smaller ones, causes emulsification, and finally the digestion of fat molecules. All bacteria have a cell wall containing fatty acids that can be destroyed by bile salts [34]. The isolates that are resistant to high concentrations of bile salts, can survive and grow in the normal concentration of bile in the human gastrointestinal tract. The secretion of bile salts into the duodenum directly disrupts the growth of probiotic bacteria. The detergent properties of bile salts enable them to penetrate into bacterial cell membranes, disrupting their structure and changing cell homeostasis [35]. The cow bile used in this study contained conjugated and non-conjugated bile salts such as taurocholic acid, glycolic acid (hydroxyacetic acid), lectin, and cholesterol. The results of the resistance of the isolates to different concentrations (0.3, 0.5, and 1%) of bile salts and non-conjugated bile salts are shown in Table 4. KK3 and KF2 were the most susceptible isolates to the bile salts. In the 0.3% of bile salts, the cell viability of KK3 and KF2 was 7.90 ± 0.14% and 8.1 ± 0.47%, respectively, and at higher concentrations, isolates did not grow.

The highest cell viability at bile salts was observed in KK4 and KF3 isolates. The resistance and sensitivity of isolates to conjugated bile salts (TC: sodium taurocholate, TDC: sodium taurodeoxycholate, GC: sodium glycolate, GDC: Sodium glicodeoxycholate) were also investigated (Table 4). The results showed that isolates of *Lactococcus lactis* RSg (KK1) and *Enterococcus faecium* KKP 3772 (KF1) were resistant against all non-conjugated bile salts and could grow. Exceptionally, *Enterococcus faecium* C1 (KF2) had weak growth in TC.

*Lpb. plantarum* PM411 (KK5) and *Pediococcus pentosaceus* H11 (KF3) were resistant to the conjugated bile salts, except for GDC, but *Pediococcus pentosaceus* H11 (KF3) did not show deconjugation activity against TC and TDC. *Enterococcus faecalis* P190052 (KK2) was resistant to all of the deconjugated bile salts and showed the ability to deconjugate GDC and TDC. *Enterococcus mundtii* CECT972T (KK3) was susceptible to all compounds and showed only weak growth in the presence of GC. *Lpb. plantarum* PM411 (KK5) was resistant in the media containing TC, TDC, and GC bile salts, but weak growth was observed in media containing GDC. Generally, among all isolates, *Enterococcus mundtii* CECT972T (KK3) and *Enterococcus faecium* C1 (KF2) were the most sensitive isolates to bile salts. While *Pediococcus pentosaceus* VNK-1 (KK4) and *Pediococcus pentosaceus* H11 (KF3) showed the highest resistance. 

### 3.3. Antibiotic Susceptibility

Table 5 displays the antibiotic resistance of bacterial isolates against 16 different antibiotics. Based on the results obtained, all isolates were resistant to tetracycline and also all of them were susceptible to chloramphenicol and Gentamicin. Most of the isolated LAB were susceptible to vancomycin and only *Lpb. plantarum* PM411 (KK5) and *Pediococcus pentosaceus* VNK-1 (KK4), showed moderate resistance. Among the tested isolates, only *Enterococcus faecium* C1 (KF2) was resistant to cefixime and sulfamethoxazole. *Pediococcus pentosaceus* VNK-1 (KK4) showed resistance only to cetracycline, cefepime, and clindamycin antibiotics. Generally, there are two main mechanisms for the antibiotic resistance of probiotics: (1) natural or intrinsic resistance, which is not transferable, (2) acquired resistance, usually caused by bacterial mutation, which might transfer plasmid encoding of antibiotic resistance genes and is potentially transferable to pathogenic bacteria or other commensals [36].

The European Federation of Animal Science (EFAS) and the European Food Safety Authority (EFSA) recommend that the strains of LAB which are used in different medicines and foods, should not have transferable antibiotic resistance genes to be considered safe for human and animal consumption [37]. Antibiotic resistance may have negative effects on probiotics. For instance, antibiotic resistance genes can be transferred to pathogenic or intestinal bacteria and make them resistant to antibiotic treatment [38,39]. Gupta and Sharma (2017) stated *Pediococcus acidilactici* Ch-2 was susceptible to 11 out of 12 antibiotics. Therefore, this isolate can be considered a safe probiotic strain [40]. 

### 3.4. Antibacterial Activity

Antimicrobial activity is one of the important characteristics to evaluate the probiotic potential of a microorganism. The antibacterial activity of probiotics can be due to the synthesis of H_2_O_2_, ethanol, phenols, diacetyl, proteins, and organic acids such as acetic and lactic acids that are produced during the growth of probiotics. These metabolites, together with help of a competitive exclusion mechanism, in which probiotics compete with harmful microorganisms for adhesive receptors and nutrients, can destroy and inhibit the colonization of pathogens in the body [41]. In this study, the effect of pH, temperature, and enzyme on antimicrobial activity was determined. The results of the antimicrobial effect of the isolates are presented in Table 6. The diameters of the inhibition zones on the culture medium of pathogen strains were measured as a criterion for measuring the antimicrobial activity of the isolates. Comparing the antimicrobial activity of different isolates showed that the antimicrobial activity of *Pediococcus pentosaceus* VNK-1 (KK4) and *Pediococcus pentosaceus* H11 (KF3) was higher than other strains and their inhibition zones were larger. *Enterococcus mundtii* CECT972T (KK3) and *Lpb. plantarum* PM411 (KK5) showed the smallest inhibition zone in different treatments.

Overall, the obtained results revealed that the isolates had different antimicrobial activities. Some isolates showed low, + (11–15 mm); while others had moderate, ++ (15–20 mm) and high +++, (>21 mm) inhibition zones. The treatment of isolates with catalase enzyme did not change, their inhibition rate except for *Lpb. plantarum* PM411 (KK5), which presented a decreased inhibition zone diameter and lower antibacterial activity. Heat treatment only decreased the antimicrobial activity of *Enterococcus mundtii* CECT972T (KK3) and *Lactococcus lactis* RSg (KK1). Comparison between untreated and treated samples in neutral pH conditions showed that the majority of the isolates did not exhibit antimicrobial activity, and only KK4, KF3, and KK5 isolates had poor inhibition activity against pathogen strains. KK4 isolate displayed antimicrobial activity against all of the examined pathogens. Generally, the highest antimicrobial activity of all isolates was observed against *Staphylococcus aureus* ATCC 25923 while the lowest activity was detected against *Pseudomonas aeruginosa* PTCC 1707.

### 3.5. Cell Surface Hydrophobicity and Auto-Aggregation

The cell surface hydrophobicity and auto-aggregation are phenotypic characteristics that are directly attributed to the adhesion ability of bacteria. Cell surface hydrophobicity is the nonspecific interaction between host and bacterial cells. Solvents such as xylene, chloroform, n-octane, n-hexadecane, and ethyl acetate are used to measure this feature [18,42]. This characteristic is supposed to be a major factor in determining the capability of LAB to adhere to the intestinal cells and their consequent proliferation [43]. The adhesion depends on some factors, such as van der Waals force, Brownian motion, electric charge of the surface, and gravitational force. Additionally, surface hydrophobicity depends on the type of bacteria, so this characteristic should be exanimated separately for each strain. The s-layer, which consists of a single molecular layer of identical proteins or glycoproteins, plays a major role in the Brownian movement of LAB [44].

**Figure 1:**
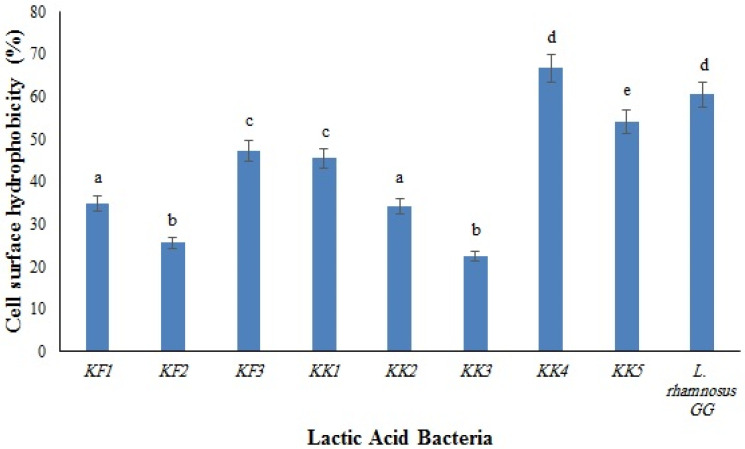
Cell surface hydrophobicity of the LAB isolates compared to the control strain, *L. rhamnosus* GG. Different letters show significant differences (*p* < 0.05).

The results of the surface hydrophobicity of the isolates are shown in Figure 1. The highest and the lowest hydrophobicity were observed in *Pediococcus pentosaceus* VNK-1 (KK4) (66.7%) and in *Enterococcus mundtii* CECT972T (KK3) (22.4%), respectively. The cell surface of microorganisms contains hydrophobic compounds such as proteins, teichoic acids, and lipids, which make them attach to the surface of the intestinal epithelium through covalent bonds. The differences between the cell surface hydrophobicity of bacteria are influenced by several parameters, such as the chemical composition and structural properties of bacteria (type of amino acids, composition of proteins, polysaccharides, and lipid compounds in the bacterial cells), the growth phase of bacteria, and environmental factors [18].

**Figure 2:**
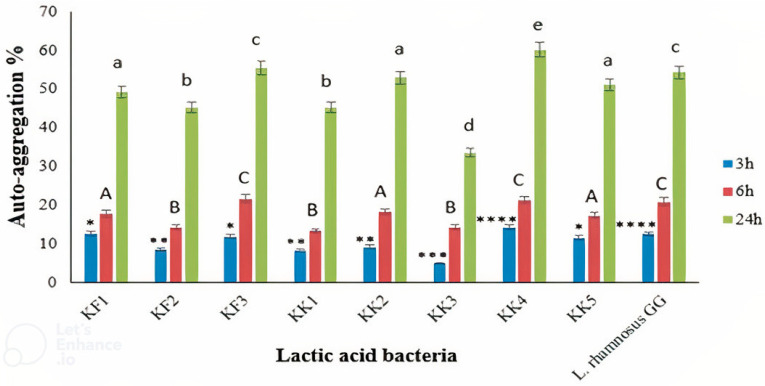
Auto-aggregation of the LAB isolates after 3, 6, and 24 h of incubation, compared to the control strain, *L. rhamnosus* GG. ∗∗∗∗ *p* < 0.0001, ∗∗∗ *p* < 0.001, ∗∗ *p* < 0.01, and ∗ *p* < 0.05. Different letters show significant differences (*p* < 0.05).

The results showed the auto-aggregation abilities of all the LAB isolates (Figure 2). After 24 h of incubation, auto-aggregation of the LAB strains was between 33.60% and 60.20%. The highest and lowest auto-aggregation were detected in *Pediococcus pentosaceus* VNK-1 (KK4) and *Enterococcus mundtii* CECT972T (KK3), respectively. The auto-aggregation rate of *Pediococcus pentosaceus* VNK-1 (KK4) (60.20%) was higher than that of *Lacticaseibacillus* GG (54.3%), as the control strain. Indicating that this bacterium probably has better cell adhesion properties than *L. rhamnosus* GG. The amount of auto-accumulation was progressively increased during 24 h of incubation. Our findings are in accordance with previous studies [21,45,46].

## 4. Conclusions

Overall, the findings of this study revealed that kashk samples from both regions (Abadeh and Kalat) contained LAB with probiotic properties which *P. pentosaceus* VNK-1 (KK4) and *P. pentosaceus* H11 (KF3) showed the best results. Therefore, they can be consumed as a native source of potentially beneficial LAB. Furthermore, the LAB strains possessing proper characteristics for acting as probiotics according to the obtained results could be used to develop new probiotic starter cultures for kashk manufacturing. Traditional fermented foods, such as kashk, have great potential to improve the nutritional quality and health of consumers. However, with the increase in urbanization and industrialization as well as the decreasing rate of acceptance of traditional foods, the consumption of traditional dairy products such as kashk has reduced, and their microbiome has been exposed to destruction. Therefore, the isolates from products such as traditional kashk with health-promoting properties as probiotics should be preserved and incorporated into food products.

## Figures and Tables

**Table 1:** Characteristics of LAB isolates of Abadeh (Fars) and Kalat (Razavi Khorasan) kashk samples.

Group Number	Region	Number of Isolates	Growth at 10 °C	Growth at 45 °C	Growth at pH 4.4	Growth at pH 9.6	Growth at 6.5% NaCl	Arginine Hydrolysis	CO_2_ Gas Production
1	Abadeh	1	+	-	+	±	+	-	-
2	Abadeh	2	+	±	±	-	+	-	-
3	Kalat	1	+	-	-	-	-	+	-
4	Kalat	4	+	+	+	+	+	+	-

**Table 2:** Identification of LAB isolates of Abadeh (Fars) and Kalat (Razavi Khorasan) kashk samples using a molecular assay.

Sampling Location	Isolate Code	Highest Similarity in NCBI Database	Similarity Percentage	Accession Number
Abadeh	KF1	*Enterococcus faecium* KKP 3772	99	OL454909
Abadeh	KF2	*Enterococcus faecium* C1	100	EU428011
Abadeh	KF3	*Pediococcus pentosaceus* H11	100	OM388463
Kalat	KK1	*Lactococcus lactis* RSg	97	KJ660075
Kalat	KK2	*Enterococcus faecalis* P190052	98	MN749533
Kalat	KK3	*Enterococcus mundtii* CECT972T	100	AJ420806
Kalat	KK4	*Pediococcus pentosaceus* VNK-1	99	ON810480
Kalat	KK5	*Lactiplantibacillus plantarum* PM411	98	JX440377

**Table 3:** The survival of LAB isolates of Abadeh (Fars) and Kalat (Razavi Khorasan) kashk samples in pH 2 and 3, as well as simulated conditions of the gastrointestinal tract (GIT) (Log CFU/mL).

	pH 2				pH 2				
Strain Code	0 h	1 h	2 h	3 h	0 h	1 h	2 h	3 h	Survival in GIT
*Enterococcus faecium* KKP 3772 (KF1)	9.05 ± 0.11	7.06 ± 0.20	6.15 ± 0.14	5.15 ± 0.05	9.11 ± 0.08	8.90 ± 0.17	9.25 ± 0.60	9.31 ± 0.22	6.28 ± 0.25
*Enterococcus faecium* C1 (KF2)	9.14 ± 0.23	6.95 ± 0.70	6.40 ± 0.15	4.65 ± 0.18	9.03 ± 0.10	9.45 ± 0.28	9.89 ± 0.26	9.46 ± 0.11	6.55 ± 0.13
*Pediococcus pentosaceus* H11 (KF3)	9.02 ± 0.14	7.22 ± 0.12	7.35 ± 0.41	6.15 ± 0.23	9.12 ± 0.16	9.32 ± 0.14	9.50 ± 0.13	9.54 ± 0.18	7.35 ± 0.11
*Lactococcus lactis* RSg (KK1)	9.12 ± 0.07	6.95 ± 0.32	5.65 ± 0.23	3.95 ± 0.12	9.09 ± 0.18	8.75 ± 0.16	8.15 ± 0.10	7.70 ± 0.39	5.45 ± 0.37
*Enterococcus faecalis* P190052 (KK2)	9.06 ± 0.19	7.02 ± 0.33	6.65 ± 0.12	6.42 ± 0.14	9.10 ± 0.13	9.51 ± 0.37	9.36 ± 0.10	9.30 ± 0.41	6.80 ± 0.16
*Enterococcus mundtii* CECT972T (KK3)	9.13 ± 0.21	7.89 ± 0.22	6.28 ± 0.15	5.67 ± 0.19	9.07 ± 0.12	9.20 ± 0.13	9.28 ± 0.34	9.18 ± 0.15	6.45 ± 0.36
*Pediococcus pentosaceus* VNK-1 (KK4)	9.03 ± 0.12	8.26 ± 0.19	7.75 ± 0.16	6.56 ± 0.35	9.05 ± 0.14	9.11 ± 0.23	9.48 ± 0.36	9.67 ± 0.41	7.52 ± 0.15
*Lactiplantibacillus plantarum* PM411 (KK5)	8.98 ± 0.31	7.23 ± 0.16	6.35 ± 0.20	5.71 ± 0.25	9.03 ± 0.15	9.10 ± 0.42	8.95 ± 0.35	8.80 ± 0.09	5.95 ± 0.20

**Table 4:** The survival of LAB isolates of Abadeh (Fars) and Kalat (Razavi Khorasan) kashk samples in the presence of different concentrations of bile (0.3%, 0.5%, and 1%), and their ability for the degradation of different bile salts.

Strain Code	Bile Acid Degradation Ability				Growth in the Presence of Bile (%, Compared to the Control Sample)		
	GDC *	GC	TDC	TC	1	0.5	0.3
KF1	g	g	g	g	7.60 ± 0.30	11.2 ± 0.42	28.50 ± 0.25
KF2	- **	-	-	wg	0	0	8.1 ± 0.47
KF3	-	g	+	+	65.04 ± 0.21	81.0 ± 0.27	84.60 ± 0.33
KK1	g	g	g	g	0	9.10 ± 0.34	17.20 ± 0.18
KK2	+	g	+	g	25.50 ± 0.30	41.40 ± 0.32	50.40 ± 0.47
KK3	-	wg	-	-	0	0	7.90 ± 0.14
KK4	wg	g	+	+	70.70 ± 0.19	82.40 ± 0.30	91.10 ± 0.42
KK5	-	g	g	g	30.20 ± 0.46	41.08 ± 0.36	63.02 ± 0.25

* GDC: sodium glicodeoxycholate; GC: sodium glycolate; TDC: sodium taurodeoxycholate; TC: sodium taurocholate; ** no growth; wg: weak growth; g: growth; +: growth and bile salt deconjugation.

**Table 5:** Antibiotic sensitivity of LAB isolates of Abadeh (Fars) and Kalat (Razavi Khorasan) kashk samples.

Strain Code	CN *	C	CRO	CC	GM	AM	E	TE	CP	K	SXT	CFM	FEB	S	CL	V
KF1	R	S	S	R	S	S	S	R	R	S	S	S	R	R	R	S
KF2	R	S	I	R	S	R	S	R	R	S	R	R	S	R	R	S
KF3	I	S	S	I	S	S	S	R	I	S	S	I	R	S	S	S
KK1	S	S	S	R	S	I	S	R	I	S	S	S	I	S	I	S
KK2	R	S	R	R	S	R	I	R	R	I	S	S	R	R	R	S
KK3	S	S	S	S	S	S	S	R	S	S	S	S	S	S	R	S
KK4	I	S	I	R	S	I	S	R	S	I	S	S	R	I	S	I
KK5	R	S	R	S	S	R	I	R	R	S	I	I	R	R	R	I

* CN: cephalexin; C: chloramphenicol; CRO: ceftriaxone; CC: clindamycin; GM: gentamicin; AM: ampicillin; E: erythromycin; TE: tetracycline; CP: ciprofloxacin; K: kanamycin; SXT: sulfamethoxazole; CFM: cefixime; FEB: Cefepime; S: streptomycin; CL: colistin; V: vancomycin. erythromycin results based on R ≤ 13 mm; I: 13–23 mm; S ≥ 23 mm. gentamicin results based on R ≤ 6 mm; I: 7–9 mm; Mm. S ≥ 10 mm. vancomycin results based on R ≤ 12 mm; I: 12–13 mm; mm S ≥ 13 mm. I: medium (zone diameter, 12.5–17.4 mm); R: resistant (zone diameter, ≥ 12.4 mm); S: susceptible (zone diameter, ≥ 17.5).

**Table 6:** Antimicrobial activity of LAB isolates of Abadeh (Fars) and Kalat (Razavi Khorasan) kashk samples.

Treatment	Indicator Pathogens	LAB							
		KF1	KF2	KF3	KK1	KK2	KK3	KK4	KK5
	*Escherichia coli* ATCC 25922	+ *	++	++	++	++	++	+++	+++
Not treated	*Staphylococcus aureus* ATCC 25923	+++	+++	+++	++	+++	++	+++	+++
	*Pseudomonas aeruginosa* PTCC 1707	+	++	++	+	++	+	+++	++
	*Salmonella typhimurium* ATCC 14028	++	++	+++	++	++	++	+++	++
	*Escherichia coli* ATCC 25922	-	-	+	-	-	-	+	+
Neutralized	*Staphylococcus aureus* ATCC 25923	-	-	+	-	-	-	+	+
	*Pseudomonas aeruginosa* PTCC 1707	-	-	-	-	-	-	+	-
	*Salmonella typhimurium* ATCC 14028	+	-	+	-	-	-	+	+
	*Escherichia coli* ATCC 25922	++	+	++	+	++	+	+++	+++
	*Staphylococcus aureus* ATCC 25923	+++	+++	+++	++	+++	++	+++	+++
Heat treatment	*Pseudomonas aeruginosa* PTCC 1707	+	++	++	+	++	-	+++	++
	*Salmonella typhimurium* ATCC 14028	++	++	+++	++	++	++	+++	++
	*Escherichia coli* ATCC 25922	++	++	++	++	++	++	+++	+
	*Staphylococcus aureus* ATCC 25923	+++	+++	+++	++	+++	++	+++	++
Catalase treatment	*Pseudomonas aeruginosa* PTCC 1707	+	++	++	+	++	+	+++	-
	*Salmonella typhimurium* ATCC 14028	++	++	+++	++	++	++	+++	+++

* The results of independent tests (3 repetitions) of inhibition zones, indicating inhibition of low, + (11–15 mm), moderate, ++ (15–20 mm), and high +++, (>21 mm).

## Data Availability

The data presented in this study are available upon request from the corresponding author.
